# Evaluating the efficacy of oxytocin for pain management: An updated systematic review and meta-analysis of randomized clinical trials and observational studies

**DOI:** 10.1080/24740527.2023.2191114

**Published:** 2023-05-15

**Authors:** Anastasia A. Mekhael, Jennifer E. Bent, Jonathan M. Fawcett, Tavis S. Campbell, Aldo Aguirre-Camacho, Alison Farrell, Joshua A. Rash

**Affiliations:** a Department of Psychology, Memorial University of Newfoundland, St. John’s, Newfoundland, Canada; b Division of Community Health and Humanities, Memorial University of Newfoundland, St. John’s, Newfoundland, Canada; c Department of Psychology, University of Calgary, Calgary, Alberta, Canada; d School of Biomedical Sciences, European University of Madrid, Villaviciosa de Odón Madrid, Madrid, Spain; e Department of Psychology, Cardenal Cisneros University College, Madrid, Spain; f Memorial University Libraries, Memorial University of Newfoundland, St. John’s, Newfoundland, Canada

**Keywords:** oxytocin, chronic pain, systematic review, meta-analysis, analgesia

## Abstract

**Background:**

There is a need for novel analgesics with favorable risk to benefit profiles. Oxytocin has recently gained attention for its potential analgesic properties.

**Aim:**

The aim of this study was to perform an updated systematic review and meta-analysis evaluating the effect of oxytocin for pain management.

**Method:**

Ovid MEDLINE, Embase, PsycINFO, CINAHL, and Clinicaltrials.gov were searched for articles reporting on associations between oxytocin and chronic pain management from January 2012 to February 2022. Studies published before 2012 that were identified in our previous systematic review were also eligible. Risk of bias of included studies was assessed. Synthesis of results was performed using meta-analysis and narrative synthesis.

**Results:**

Searches returned 2087 unique citations. In total, 14 articles were included that reported on 1504 people living with pain. Results from meta-analysis and narrative review were mixed. Meta-analysis of three studies indicated that exogenous oxytocin administration did not result in a significant reduction in pain intensity relative to placebo (*N* = 3; *n* = 95; *g* = 0.31; 95% confidence interval [CI] −0.10, 0.73). Narrative review provided encouraging evidence that exogenous oxytocin administration reduced pain sensitivity among individuals with back pain, abdominal pain, and migraines. Results suggested that individual difference factors (e.g., sex and chronic pain condition) may influence oxytocin-induced nociception, but the heterogeneity and limited number of studies identified precluded further investigation.

**Discussion:**

There is equipoise for the benefit of oxytocin for pain management. Future studies are imperative and should undertake more precise exploration of potential confounds and mechanisms of analgesic action to clarify inconsistency in the literature.

## RÉSUMÉ

**Contexte:** Il existe un besoin de nouveaux analgésiques présentant un profil de risque/bénéfice favorable. L’ocytocine a récemment attiré l’attention pour ses propriétés analgésiques potentielles.

**Objectif:** L’objectif de cette étude était d’effectuer une mise à jour d’une revue systématique et une méta-analyse pour évaluer l’effet de l’ocytocine pour la gestion de la douleur.

**Méthode:** Des recherches ont été effectuées dans Ovid MEDLINE, Embase, PsycINFO, CINAHL et Clinicaltrials.gov pour y repérer des articles sur les associations entre l’ocytocine et la prise en charge de la douleur chronique de janvier 2012 à février 2022. Les études publiées avant 2012 qui ont été recensées dans notre revue systématique précédente était également admissibles. Le risque de biais des études incluses a été évalué. Une synthèse des résultats a été réalisée à l’aide d’une méta-analyse et d’une synthèse narrative.

**Résultats:** Les recherches ont permis de recenser 2 087 citations uniques. Au total, 14 articles portant sur 1 504 personnes vivant avec la douleur ont été incluses. Les résultats de la méta-analyse et de l’examen narratif ont été mitigés. Une méta-analyse de trois études a révélé que l’administration d’ocytocine exogène n’avait pas entraîné de réduction significative de l’intensité de la douleur comparativement au placebo (N = 3; n = 95; g = 0,31; Intervalle de confiance à 95 % [IC] −0,10 ; 0,73). L’examen narratif a fourni des preuves encourageantes que l’administration d’ocytocine exogène avait réduit la sensibilité à la douleur chez les personnes souffrant de maux de dos, de douleurs abdominales et de migraines. Les résultats indiquent que les facteurs de différence individuels (par exemple, le sexe et la douleur chronique) peuvent influencer la nociception induite par l’ocytocine, mais le nombre limité d’études recensées et leur hétérogénéité a empêché d’approfondir l’enquête.

**Discussion:** Il existe un équilibre au profit de l’ocytocine pour la prise en charge de la douleur. Il est primordial que d’autres études soient menées afin d’explorer de manière plus précise les facteurs de confusion et les mécanismes de l’action analgésique potentiels et ainsi clarifier l’incohérence dans la littérature.

## Introduction

Canadian estimates suggest that approximately 15% of children^1^ and 20% of adults^2^ live with persistent pain, including 25% to 65% of community-dwelling seniors.^3^ Historically, chronic pain has been noted as one of the most difficult conditions to treat,^4^ resulting in the widespread prescription of pharmacological interventions^5,6^; these include anti-inflammatory agents, opioid analgesics, adjuvant analgesics (e.g., antidepressants or anticonvulsants), and over-the-counter medications, such as nonsteroidal anti-inflammatory drugs. The prescription of opioids has dramatically increased over the last several decades,^7^ and opioid prescription practices have been linked to the increased rates of opioid-related overdoses and adverse events in Canada.^8,9^ While carrying considerable risk of misuse and adverse effects, the effect of opioids on pain and function are modest at best.^10^ Results on the effectiveness of treatment with opioids are also inconsistent; patients often report little improvement in physical function, emotional functioning, and health-related quality of life.^6^ This points to the need for an analgesic that is nonaddictive, has few adverse effects, and is effective at reducing pain across chronic pain conditions.

### Oxytocin as a Treatment for Pain

Oxytocin is a neuropeptide that is synthesized in the paraventricular and supraoptic nuclei of the hypothalamus and released into the bloodstream through the central and peripheral nervous systems.^11^ Oxytocin is naturally released during skin-to-skin contact, massage, and lactation, and this has been reported to improve mood, decrease anxiety, and buffer self-report and physiological indicators of stress.^12,13^ Evidence suggests that oxytocin plays a role in the experience of pain.

Potentially complementary mechanisms exist through which the oxytocinergic system influences the perception of pain. Oxytocin released into the central nervous system is thought to play an important role in the modulation and transmission of pain signals.^14,15^ Animal models have indicated that oxytocin in regions of the brain such as the periaqueductal gray influence pain modulation through endogenous opiate peptides, an effect that is attenuated with the administration of oxytocin or opiate receptor antagonists.^16^ Similarly, the paraventricular spinal pathway projects oxytocin to the lamina of the dorsal horn,^17,18^ a structure in which most nociceptive primary afferent neurons terminate.^19^ Oxytocin in the dorsal horn activates a set of glutamatergic interneurons that result in GABAergic inhibition of pain transmitting Aδ- and C-fibers at nociceptive-specific and wide dynamic range neurons.^14,20,21^ There is also evidence that oxytocin is released from the supraoptic nuclei of the hypothalamus into the periphery, where it has indirect antinociceptive effects.^22^ Importantly, oxytocin does not cross the blood–brain barrier, with an estimated perfusion of 1% to 2%,^23^ and peripheral oxytocin seems unlikely to exhibit central effects.

Oxytocin may influence the experience of chronic pain indirectly through the modulation of stress and emotional states. Chronic pain is accompanied by mental comorbidities across the life span.^24–26^ Patients experiencing persistent pain across 15 primary care facilities across the world were 4.14 times more likely to be diagnosed with depressive or anxiety disorders meeting *International Classification of Diseases*, Tenth Revision diagnostic criteria relative to those without chronic pain.^27^ Evidence suggests that astrocytes in the central amygdala express oxytocin receptors and mediate anxiolytic effects within an animal model of chronic neuropathic pain.^28^

### Rationale

Rash et al.^29^ conducted the first systematic review of the literature on oxytocin and pain, including human and animal studies published between 1950 and 2012. It was reported that oxytocin increased pain tolerance in the majority of studies that met inclusion criteria and that this effect was consistent across different modes of administration (e.g., intravenous, intranasal) and in response to diverse noxious stimuli (e.g., electric or heat). The authors concluded that the use of oxytocin as an analgesic for acute pain in animals was supported. Moreover, preliminary research suggested that oxytocin could also decrease pain sensitivity among humans. A call was made for additional methodologically rigorous research among human populations before definitive conclusions could be drawn regarding the effects of oxytocin on pain among humans.^29^ As such, the goal of the present review is to update the systematic review performed by Rash et al.^29^ This review is necessary given that a large body of mixed results exists around the effects of exogenous oxytocin on pain sensitivity.^30–32^ Contradictory findings also exist in the literature evaluating the association between oxytocin and emotional functioning.^33,34^ Moreover, there have been additional studies published since 2012 that would strengthen our understanding of the interplay between oxytocin and pain. Finally, an updated systematic review will identify gaps in the literature, highlight areas for future research, and enhance our understanding of pathophysiological mechanisms involved in the experience of pain.

## Methods

### Questions to be Addressed

Three questions were triangulated to better understand the role of the oxytocinergic system in nociception among individuals with chronic pain: (1) Is there a reliable effect of exogenous oxytocin administration on sensitivity to pain among people with chronic pain? (2) Is there a reliable inverse association between oxytocin concentration and pain reported among individuals who experience chronic pain? and (3) Do basal oxytocin concentrations differ among individuals with chronic pain and healthy controls? Similar evidence was triangulated to better understand the effect of oxytocin on secondary outcomes of emotional function among individuals who experience chronic pain.

### Protocol and Registration

The protocol was preregistered on the Prospective Register of Systematic Reviews (PROSPERO No. CRD42021234926). This systematic review and meta-analysis was prepared in accordance with the PRISMA (Preferred Reporting Items for Systematic Review and Meta-analyses) guidelines; refer to Supplemental Table 1 to view the PRISMA checklist.^35^

### Study Eligibility Criteria

#### Population

Studies including human participants with a primary diagnosis of chronic noncancer pain were flagged for inclusion.^36^ No restriction was placed on type or location of pain. Given that chronic pain is prevalent across the life span,^37,38^ studies were eligible for inclusion regardless of age of participants included.

#### Intervention

To evaluate the effect of exogenous oxytocin administration on pain and function, we intentionally cast a broad net and considered studies that administered oxytocin exogenously for inclusion, regardless of route of administration (i.e., peripheral or central), dose of oxytocin delivered, or frequency of administration.

Observational studies without an intervention were eligible for inclusion if they reported associations or comparisons that permitted an evaluation of the role of the oxytocinergic system on pain.

#### Comparison

When evaluating the effect of exogenous oxytocin administration on pain, studies that contained an active comparison condition (e.g., placebo, intravenous administration without oxytocin) were eligible for inclusion. Studies were eligible for inclusion if they reported on pain occurring throughout the day or pain in response to an acutely painful procedure.

When evaluating the association between oxytocin and reports of pain, studies that reported on the association between oxytocin and pain among people with chronic pain were eligible.

When evaluating differences in basal oxytocin concentrations among people who experience chronic pain and healthy controls, studies that included a comparison of basal endogenous oxytocin levels between people who experience chronic pain and healthy controls were eligible.

#### Outcomes

In alignment with the Initiative on Methods, Measurement, and Pain Assessment in Clinical Trials,^39^ the primary outcomes of interest were pain and physical function. We did not restrict studies based on the method used to quantify our primary outcomes (e.g., self-report using numeric or visual analogue scales, remission status, etc.) due to the novelty of this area of investigation. Secondary outcomes included emotional function (e.g., depressed or anxious mood) and adverse effects.

#### Design

Controlled, noncontrolled, and observational studies were eligible for inclusion to gain a more comprehensive understanding of the association between the oxytocinergic system and pain and to assess its efficacy as a potential analgesic.

### Study Exclusion Criteria

Studies were excluded if they reported on patients with chronic pain related to cancer, because the conditions may have different origins^40^ and comorbidities, and treatment goals may differ depending on patient prognosis. Studies that reported on labor and birth-related pain were also excluded, because oxytocin is typically used to induce labor^41^ and may be present in higher-than-normal levels during childbirth.^42^ Studies that included participants who have had a portion of the brain removed were also excluded, because these surgeries may have effects on pain perception that obscure potential relationships between oxytocin and pain.^29^ Studies that reported on one case or a small series of cases (i.e., *n* < 3) were excluded. Finally, studies were excluded that delayed pain testing for longer than 3 h after oxytocin administration to ensure maximum concentration upon measurement of pain given that central oxytocin concentration peaksbetween 30 and 60 min after exogenous administration.^43^

### Data Sources and Search Strategy

A preliminary search strategy was created with the guidance of a health science librarian (A.F.). An independent information specialist then peer-reviewed the strategy prior to implementation using the Peer Review for Electronic Search Strategies checklist.^44^ We searched four bibliographic databases from 2012 to February 14, 2022: (1) Ovid MEDLINE, excluding indexed citations for conference abstracts and posters; (2) Embase (Embase.com); (3) PsycINFO; and (4) CINAHL. The Clinicaltrials.gov website was also searched for ongoing studies of potential relevance. Studies published before 2012 that were identified in the previous systematic review by Rash et al.^29^ were eligible for inclusion if they met all inclusion criteria. Finally, records were obtained through hand searches. The full search strategy for Ovid Medline can be found in the protocol (PROSPERO No. CRD42021234926).

### Data Collection and Analyses

#### Study Screening

Searches were conducted, duplicates were removed using Endnote X9,^45^ and results were imported into the Covidence^46^ online citation manager for systematic reviews. Two independent reviewers (A.A.M., J.E.B.) screened search results against eligibility criteria using a two-step procedure: (1) title and abstract screening and (2) potentially relevant papers retrieved for full-text screening. Disagreements between reviewers were resolved through discussion or mediation by J.A.R. Agreement between reviewers was calculated using percentage agreement.^47^

#### Data Extraction

Data extraction was completed using a predefined rubric that captured the following information during full-text review: (1) journal article information (i.e., author’s names, country, journal, DOI, publication year), (2) methodological information (i.e., design, method of oxytocin administration or assessment, standardized and study-specific measures, duration, potential shortcomings/limitations in the methodology, and type of comparison), (3) sample characteristics (i.e., sample size, age, sex, recruitment, unique sample characteristics, type of chronic pain, history with chronic pain), and (4) results (e.g., means and standard deviations reflecting change in pain and function, correlation between oxytocin measurement and pain or function, missing data). Extraction was compared across raters to ensure accuracy. Discrepancies were resolved by discussion or arbitration by J.A.R. Study authors were contacted via e-mail when additional information was required. Authors who did not respond were provided with two reminder e-mails before information was considered unavailable due to nonresponse.

#### Risk of Bias Assessment

Consistent with recommendations,^48^ risk of bias was considered within domains that reflect aspects of study conduct that have been reliably associated with bias among trials and observational studies.^49–51^ Risk of bias for randomized controlled trials (RCTs) was assessed using the Critical Appraisal Skills Program tool for RCTs.^52^ The domains assessed included randomization, blinding of participants, investigators and outcome assessors, attrition and handling of missing data, equivalence among participants at pretreatment, and reporting precision of estimated effects. Nonrandomized controlled studies were assessed using this tool with items pertaining to randomization omitted. Risk of bias in observational studies was assessed using the Critical Appraisal Skills Program appraisal tool for cohort studies.^53^ Domains assessed included risk of bias in recruitment, accuracy of exposure, risk of bias in assessing outcomes of interest, and the identification and handling of potential confounds. Methodological quality and risk of bias assessment was conducted independently by two reviewers (A.A.M., J.E.B.). Discrepancies were resolved by consensus or arbitration by J.A.R.

### Approaches to Evidence Synthesis

#### Quantitative Synthesis

Random effects meta-analysis was performed using the DerSimonian and Laird estimation method performed with Comprehensive Meta-Analysis software^54^ in cases where three or more studies reported on the same outcome measured in a similar manner ^55^ using the DerSimonian and Laird estimation method. Studies included in meta-analyses were categorized according to study design and outcome variables; refer to Table 1. Data were not pooled across randomized controlled trials and observational studies. Effect size calculations were performed using formulae reported in Lipsey and Wilson.^56^

**Table 1.** Characteristics of included studies arranged by similarity in outcomes and analysis undertaken.

Study	Type of chronic pain assessed (main pain outcome)	Method of oxytocin assessment	Method of pain assessment	Control	Sample size included in original analysis (mean age)		% Female	% Affected by mood disorder	Analysis of emotional functioning
Observational studies (included in meta-analysis and narrative review)									
					Controls	Treatment			
Flynn et al.^68^	Chronic pelvic pain (improvement in pain and function)	Daily administration of 24 IU intranasal OT for 2 weeks	Daily diaries of BPI-SF	Crossover arm, randomized to placebo	PBO: 12 (37.7)^b^	12 (37.7)	100	75	Narrative synthesis using the DASS
Mameli et al.^70^	Fibromyalgia (improvement in pain)	Daily administration of 40 IU of intranasal OT for 1 week then 80 IU for 2 weeks	VASPI	Independent samples, randomized to placebo	PBO: 14 (51.9)^b^	14 (51.9)	100	64.23	Narrative synthesis using Zung Self-Rating Depression Scale
Ohlsson et al.^71^	Chronic constipation (improvement in pain)	Twice-daily administration of 24 IU of intranasal OT	GSRS	Crossover arm, randomized to placebo	PBO: 26 (49)^b^	23 (47)	100	23.08 PBO; 39.13 TRT	Narrative synthesis using PGWB
Randomized controlled trials (not included in meta-analysis)									
					Controls	Patients			
Boll et al.^65^	Chronic low back pain (acute heat pain task)	Single administration of 24 IU intranasal OT	VASPI	HCs recruited with a crossover design	HCs: 22 (34.41)	22 (36.82)	0	N/A	N/A
Tracy et al.^72^	Chronic neck and shoulder pain (acute heat pain task)	Single administration of 24 IU intranasal OT	Pain intensity rated on 11-point NRS	HCs recruited. crossover design	HCs: 24 (28.46)	24 (28.46)	33% in each group	N/A	N/A
					Controls	Patients			
Louvel et al.^69^	Irritable bowel syndrome (acute pain task)	Two consecutive intravenous administrations of 10, 20, 30 or 50 µU/min OT (10 h apart)	Pain threshold during isobaric colonic distension	Repeated measures design	N/A	26 (45)	42.3	N/A	N/A
Yang^74^	Chronic low back pain (improvement in pain). Basal plasma OT was also measured	Two intrathecal (0, 0.1, 0.2, 0.4, 0.8, 1.6 µ/kg) or intravenous (50, 100, 200, 400 µg/kg) administrations of OT	Complete, partial, or no relief rated using self-report	HCs recruited for basal OT level comparison; subset of patients received placebo to control for OT effects	NR	608 (47.2) *N*_OT(ith)_ = 337 *N*_PBO(ith)_ = 77 *N*_OT(IV)_ = 151 *N*_PBO(IV)_ = 43. Breakdown of age not reported	38.3% of patients	N/A	Examined but not reported in study
Wang et al.^73^	Chronic headache and migraine (improvement in pain). Baseline OT was measured from plasma and CSF	Intranasal (100, 200, or 400 µg/kg) administrations of OT	Complete, partial, or no remission rated using self-report	HCs recruited and placebo administered	HCs: 103 (45.6)	112 (44.5)	59.22% (CTRLs); 56.25% (PTs)	N/A	N/A
Observational studies (included in meta-analysis and narrative review)									
					Controls	Patients			
Anderberg and Uvnäs-Moberg^63^	Fibromyalgia	Fasting morning plasma levels of OT assessed twice (14 days apart)	Daily pain ratings correlated with levels of OT	HCs	HCs: 30 (mean age not provided)	39 (48.6)	100% in both groups	0 (HCs) 17 (PTs)	Correlations examined qualitatively
Alfvén^61^	Pediatric chronic abdominal pain	Fasting morning plasma levels of OT assessed	VAS ratings of pain intensity, frequency, and duration correlated with OT levels	HCs	HCs: 79 (10.9)	*N*_TOT_ = 48 (9.6) *N*_RAP-P_ = 32 *N*_RAP-N_ = 9 *N*_FU_ = 25 *N*_IBD_ = 15	62.03% (HCs); 75% (PTs)	N/A	N/A
Alfvén et al.^62^	Pediatric chronic abdominal pain	Fasting morning plasma levels of OT assessed	Plasma OT levels compared across controls and patients	HCs	HCs: 34 (11)	40 (10)	55.88% (HCs); 47.5% (PTs)	N/A	N/A
Clark et al.^67,a^	Fibromyalgia (improvement in pain)	Measured salivary oxytocin levels pre and post animal-assisted therapy or control therapy	NRS taken pre- and posttreatment	Patients randomized to non-animal-assisted therapy control group	PBO: 110 (43.99)	111 (43.03)	92.73% (CTRLs); 91.89% (PTs)	N/A	Correlations examined qualitatively
Bazzichi et al.^64,a^	Fibromyalgia (improvement in pain)	Measured plasma oxytocin pre and post balneotherapy vs. mud bath therapy	VAS taken pre- and posttreatment	No HCs. Patients randomized to mud bath therapy (i.e., not balneotherapy)	21 (52.81)	20 (54.00)	95.24% (CTRLs); 95% (PTs)	7 (CTRLs); 4 (PTs)	N/A
Boström et al.^66,a^	Treatment-refractory EM and CM (improvement in chronic pain condition)	Measured salivary oxytocin levels pre- and posttreatment (i.e., vagus nerve stimulation)	VAS taken pre- and posttreatment	Age- and sex-matched HCs	14 (46.9); only 12 included in analyses	12 (47.6)	100% (CTRLs); 100% (PTs)	0 (CTRLs); 9 (PTs)	Examined qualitatively

Forty IE International Einheit of OT is equivalent to 24 IU. Percentage of participants affected by a mood disorder refers to a clinically significant/diagnosed mood disorder.

^a^The study was an RCT, but only data from observational assessments were included.

^b^Crossover trial.

BPI-SF = Brief Pain Inventory-Short Form; CM = chronic migraine; CSF = cerebrospinal fluid; CTRL = control; DASS: Depression Anxiety Stress Scale; EM = episodic migraine; FU = follow-up; GSRS = Gastrointestinal Symptoms Rating Scale; HC = healthy control; IBD = irritable bowel disease; ith = intrathecal; IU = international units; IV = intravenous; N/A = not available (denotes information that was not reported); NR = not reported; NRS = numeric rating scale; OT = oxytocin; PBO = placebo; PGWB = Psychological General Well-Being Index; PT = patient; RAP-N = recurring abdominal pain of nonpsychosomatic origin; RAP-P = recurring abdominal pain of psychosomatic origin; TOT = total; TRT = treatment; VAS = visual analogue scale; VASPI = visual analogue scale of pain intensity.

##### Mean Differences

The effect size convention for studies reporting on differences between means was the standardized mean difference. Differences between means were calculated for each trial arm (e.g., oxytocin or placebo) using raw means and standard deviations (SDs). When presented, median and interquartile ranges were converted to means and SDs using the formula recommended in the *Cochrane Handbook* (Mean ≅ Median and SD ≅ (Interquartile range)/1.35).^75^ Standard deviations of mean differences were calculated according to the formula where

$$SD{\;}_{change}=\;\left(\sqrt{\left[\begin{array}{c} SD_{baseline}^{2}+SD_{final}^{2}\\ -\left(2\times Corr\times SD_{baseline}\times SD_{final}\right) \end{array}\right]}\right)$$

Moderate correlations (i.e., *r* = 0.3) were used to calculate the change in standard deviation for each condition given that sensitivity analyses with small, moderate, and high correlations of 0.1, 0.3, and 0.5 did not result in appreciable differences. Effect sizes were calculated such that positive values indicated an effect favoring oxytocin.

##### Association between Oxytocin and Pain

The effect size convention used for studies reporting on strength of association was quantified using Spearman’s correlation, *p*. We chose the Spearman’s correlation given that it accounts for the rank order of the correlation as well as monotonic relationships between variables. Patient-level data or correlation coefficients derived from patient-level data were obtained directly from authors via e-mail when not provided in articles.

##### Assessment of Heterogeneity

Heterogeneity was assessed using *I*^2^ and prediction intervals. *I*^2^ is a measure of the proportion of overall variability in the reported effect attributable to “true” differences between studies relative to the variation attributable to sampling error. *I*^2^ has arbitrary benchmarks pertaining to small, moderate, or high levels of heterogeneity and for this reason has become prevalent in meta-analyses.^57^ Prediction intervals (PIs) were calculated to indicate the degree to which the effect differs across included studies and to provide a measure of the range of effects expected if a well-powered study were to be conducted and included in the current model.^57^

##### Assessment of Practical Significance

Statistically, practical significance for group differences was estimated by evaluating the pooled effect size against the recommended minimum effect size representing a practically significant effect for social science data, *g* = 0.41.^58^

##### Publication Bias

Publication bias was not evaluated given the small number of studies that met inclusion criteria and were able to be pooled.

#### Narrative Synthesis

Narrative synthesis was performed for outcomes where quantitative synthesis was deemed inappropriate because (1) there was significant methodological heterogeneity,^59^ (2) the number of included studies precluded quantitative synthesis (i.e., fewer than three studies), and (3) effect estimates could not be calculated due to insufficient available data.

Outcomes from studies synthesized using narrative review were reported according to the Synthesis Without Meta-analysis guideline.^60^ Results from narrative synthesis included a rationale for the grouping of studies to be synthesized and a standardized metric for intervention effects (i.e., individual effect sizes for each study with the associated 95% confidence interval).^56^ The standardized mean difference (i.e., Cohen’s *d*) was calculated for studies that compared means. Odds ratios were computed for studies that reported frequency data, and Spearman’s correlation was computed as an effect size for degree of association. Heterogeneity was evaluated based on degree of methodological diversity,^59^ including outcomes of interest and modality of the intervention.

## Results

### Study Identification

**Figure 1.**
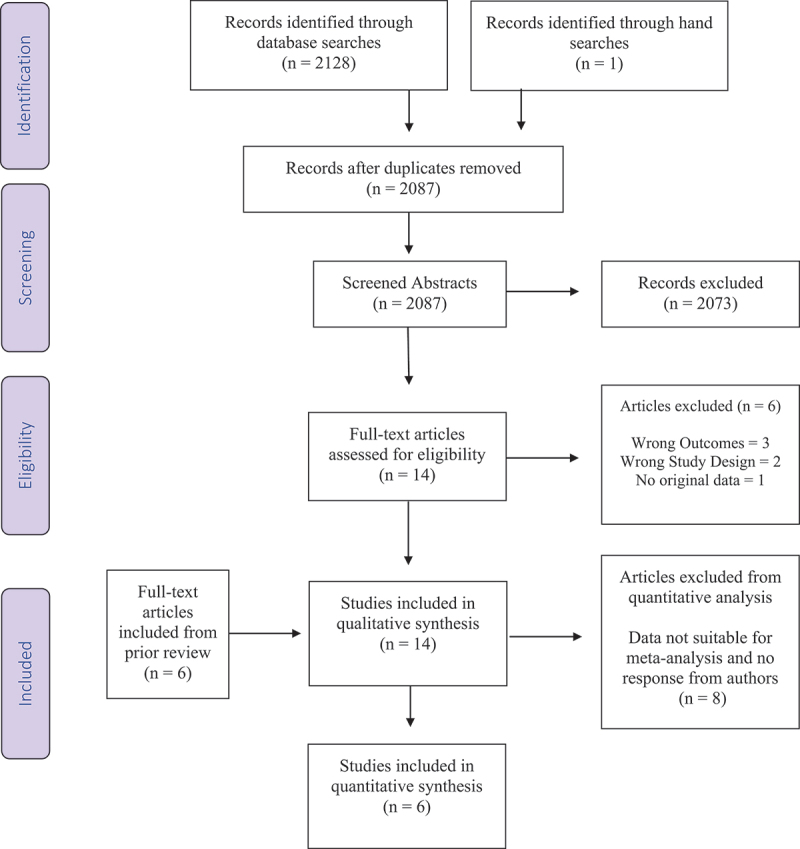
PRISMA flow chart of studies included and excluded throughout each phase of the systematic review.

Database and hand searches returned 2087 unique citations, of which 14 proceeded to full-text review. Proportion of agreement between independent reviewers during the abstract screening stage was 97.68%, indicating substantial agreement. In total, eight citations met full inclusion criteria, with an additional six articles identified in our previous review^29^ being included, for a total of 14 articles.^61–74^ Figure 1 presents a flow diagram depicting citation screening.

### Study Characteristics

Table 1 provides a summary of the included studies with relevant information related to sample characteristics, study design, methodology pertaining to pain and oxytocin assessment, and outcomes of interest. Sample sizes ranged between 12 and 608 participants, with data from 1504 participants in total. Five studies focused on adult women, two of which assessed fibromyalgia,^63,70^ one chronic pelvic pain,^68^ one chronic constipation,^71^ and one chronic migraine.^66^ One study recruited adult males who experienced chronic low back pain.^65^ Seven studies collected data from men and women with diverse chronic pain conditions (i.e., chronic neck and shoulder pain^72^; chronic low back pain^74^; fibromyalgia^64,67^; irritable bowel syndrome^69^; chronic migraine^73^). The remaining two studies examined recurrent abdominal pain among boys and girls.^61,62^

Of the 14 included studies, 6 were RCTs that involved the exogenous administration of intranasal oxytocin.^65,68,70–73^ Two studies administered exogenous oxytocin by intravenous infusion^69,74^ and one intrathecally.^74^ Outcomes of interest varied, such that 3 RCTs evaluated improvement in self-reported pain using visual analogue or numeric rating scales.^68,70,71^ Two RCTs evaluated complete, partial, or no pain relief.^73,74^ Three studies assessed sensitivity to acute pain stimuli after exogenous oxytocin administration in patients and healthy controls.^65,69,72^ Finally, 6 of the included studies were observational^61–64,66,67^ and evaluated the associations between endogenous oxytocin and pain or emotional functioning. Note that Clark et al.,^65^ Bazzicchi et al.^62^ and Boström et al.^64^ conducted RCTs on interventions that did not pertain to oxytocin and were classified as observational studies in this review given that only baseline data were used to quantify degree of association.

Mean duration of illness was only reported in six studies and ranged between 4.5 and 11.9 years.^63,64,68,70,73,74^ Six studies reported measuring emotional functioning.^63,66–68,70,71^ Yang reported insufficient data to calculate effect sizes for inclusion.^72^

### Risk of Bias

Table 2 depicts a summary of risk of bias assessment for the eight included RCTs and the six included observational studies. Overall risk of bias was low, with few studies being flagged for low methodological rigor. Most trials adequately reported on randomization (six out of eight), adjustment for attrition (six out of eight), blinding of patients (eight out of eight), blinding of investigator (seven out of eight), and baseline equivalency (six out of eight). No trials blinded outcome assessor (zero out of eight), and few reported the precision of estimates in treatment effects (four out of eight).

**Table 2.** Risk of bias of included studies.

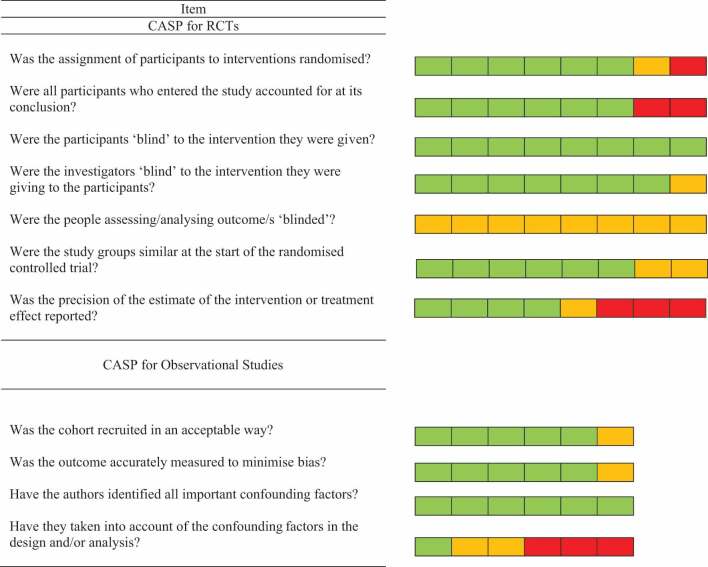

Items answered as “yes,” “can’t tell,” and “no” are represented by green, yellow, and red squares, respectively. Eight randomized controlled studies and 6 observational studies were included.

There was low risk of sampling bias across most observational studies (five out of six). All observational studies identified pertinent potential confounds (six out of six), but few (two out of six) took such confounds into account in statistical analysis or design. Most observational studies measured outcomes in a valid and reliable manner (five out of six).

#### Quantitative Synthesis

##### Effect of Exogenous Oxytocin on Pain Intensity

**Figure 2.**
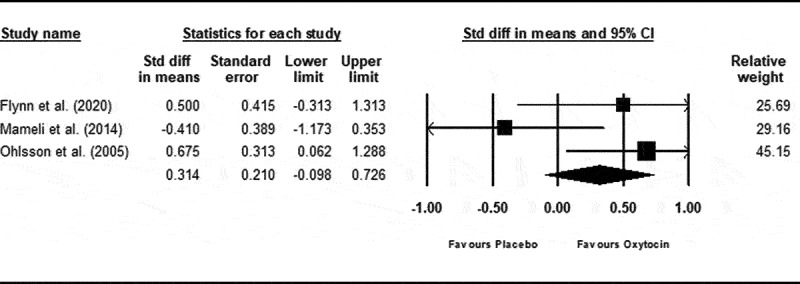
Change in self-reported pain ratings after oxytocin or placebo administration. Positive values represent a change favouring oxytocin.

Three RCTs evaluated the administration of exogenous oxytocin (i.e., intranasal oxytocin) and were pooled in meta-analysis.^68,70,71^ As depicted in Figure 2, results from the random effects model yielded a nonsignificant pooled effect (*g* = 0.31; 95% CI −0.10, 0.73; 95% PI −1.89, 2.48), favoring oxytocin. Statistically significant heterogeneity was observed between effects (*Q*_df = 2_ = 4.81; *P* = 0.09; $\tau^{2}=0.192$; *I*^2^ = 58.46%; 95% CI 0%, 88.17%).

##### Association between pain ratings and Endogenous Oxytocin Levels

**Figure 3.**
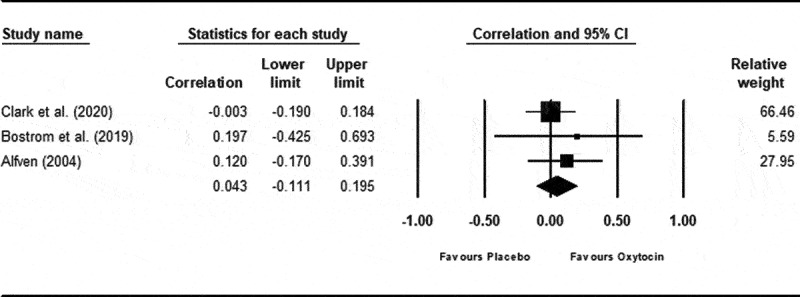
Correlation between self-reported ratings of pain intensity and basal oxytocin concentration.

Results pertaining to degree of association between pain ratings and basal endogenous oxytocin levels were pooled across one study that measured plasma oxytocin^61^ and two studies that measured oxytocin in saliva.^66,67^ There was a small nonsignificant association between peripheral oxytocin levels and self-reported pain ratings (*p*_pooled _= 0.04; 95% CI −0.11, 0.20; 95% PI −2.91, 0.48; *P* = 0.59; *Z* = 0.54; refer to Figure 3). There was no evidence of statistical heterogeneity (*Q*_df =2_ = 0.719; *P* = 0.70; $\tau^{2}=\;0$; *I*^2^ = 0%; 95% CI 0%, 71.06%).

#### Secondary Outcomes

##### Association between Depressed Mood and Peripheral Endogenous Oxytocin Levels

**Figure 4.**
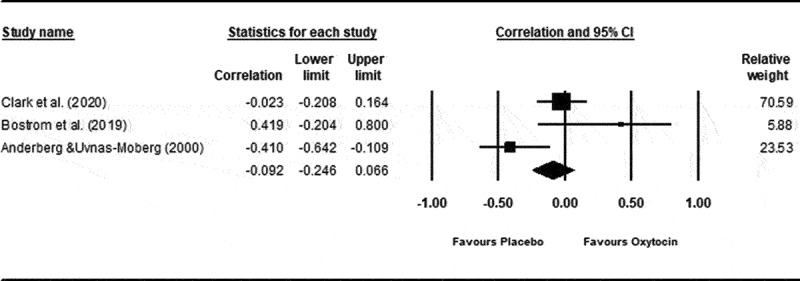
Correlation between self-reported ratings of depressed mood and basal oxytocin concentration.

Three studies provided sufficient data to conduct one meta-analysis evaluating the association between peripheral basal endogenous oxytocin levels and depressed mood.^63,66,67^ There was a small negative correlation between self-report measures of depressed mood and peripheral endogenous oxytocin concentration that was not statistically significant (*p*_pooled_ = −0.08; 95% CI −0.43, 0.30; 95% PI −2.03, 1.69; *P* = 0.70; *Z* = −0.39; refer to Figure 4). There was evidence of statistically significant heterogeneity (*Q*_df = 2_ = 7.378; *P* = 0.025; *I*^2^ = 72.89%; 95% CI 8.83, 91.94).

### Narrative Synthesis

Table 3 depicts studies that were narratively synthesized, grouped according to outcomes of interest.

**Table 3.** Narrative synthesis.

Study	Outcome	Type of effect-size	Effect size^a^ (95% CI)
Primary outcomes			
Boll et al.^65^ Tracy et al.^72^ Louvel et al.^69^	Pain intensity (Boll, Tracy) and pain threshold (Louvel)	Individual effect sizes	Boll: *d* = 0.57 (−0.02, 1.16) Tracy: *d* = −0.16 (−0.72, 0.41) Louvel: *d = *1.21 (0.50, 1.29)
Yang^74^ Wang et al.^73^	Presence of pain relief (Yang, Wang)	Odds ratio at the mid-level dosage of intravenous and intrathecal OT	Yang: Intrathecal; OR = 709.33 (70.81, 7105.33) Intravenous; OR = 1 (0.06, 16.52) Wang: Intranasal; OR = 27.44 (5.31, 141.82)
Secondary outcomes			
Flynn et al.^68^ Mameli et al.^70^ Ohlsson et al.^71^	Change in depressed mood	Mean difference of the mean differences between depression ratings in OT and PBO (i.e., [post-OT − pre-OT] − [post-PBO − pre-PBO])	Flynn: *d* = 0.22 (−0.58, 1.02) Mameli: *d* = 0.05 (−0.71, 0.80) Ohlsson: *d* = −0.16 (−0.76, 0.44)
Flynn et al.^68^ Mameli et al.^70^ Ohlsson et al.^71^	Change in anxious mood	Mean difference of the mean differences between anxiety ratings in OT and PBO (i.e., [post-OT − pre-OT] − [post-PBO − pre-PBO])	Flynn: *d* = 0.09 (−0.72, 0.88) Mameli: *d* = −0.13 (−1.13, 0.39) Ohlsson: *d* = −0.15 (−0.45, 0.75)
Clark et al.^67^ Anderberg and Uvnäs-Moberg^63^	Association between anxiety and OT levels	Separate correlations between self-reported anxiety ratings on a 10-point scale and OT levels in patients for each study	Clark: *r* = 0.02 Anderberg: *r* = −0.46

^a^Effect sizes calculated such that positive values favor the effect of oxytocin.

OT = oxytocin; PBO = placebo; Post = self-report ratings of pain, anxiety, and depression after oxytocin administration; Pre = self-report ratings of pain, anxiety, and depression before oxytocin administration.

#### Effect of Exogenous Oxytocin on Acute Pain Sensitivity

Studies that used acute pain tasks (e.g., heat pain thermode) to evaluate pain sensitivity were grouped for comparisons. Louvel et al.^69^ reported on pain threshold and was grouped with Boll et al.^65^ and Tracy et al.,^72^ who reported on pain intensity. These outcomes were evaluated together given that (1) participants reported an average of 7 to 8 out of 10 on a numeric rating scales of pain before reaching pain tolerance^76^ and (2) a validation study on pain severity and pain threshold reported no significant differences in pain severity among patients with chronic pain with high or low pain tolerance scores.^77^

Two of the three included studies that evaluated the effect of oxytocin on acutely painful procedures supported the use of oxytocin as an analgesic. Boll et al.^65^ used a finger span device while a thermal stimulus was applied to the lower back via thermode to elicit pain. The results indicated a moderate effect size (*d* = 0.57; 95% CI −0.02, 1.16), with oxytocin decreasing pain perception in patients with chronic back pain. It is also noteworthy that authors reported a significant interaction of group by substance, in that oxytocin decreased pain perception in patients with chronic pain but not in healthy controls. Similarly, Tracy et al.^72^ applied a noxious thermal stimulus to three different sites on the neck and shoulder region to elicit pain. The results showed a small, nonsignificant effect size, favoring placebo (*d = −0*.16; 95% CI −0.72, 0.41). Pain ratings were lower in the placebo condition for patients with chronic pain, whereas pain ratings were lower in the oxytocin condition among healthy controls. Finally, Louvel et al.^69^ monitored pain threshold during isobaric distension at different doses of oxytocin and placebo. A large effect size was computed (*d =* 1.21; 95% CI 0.50, 1.29) at the median oxytocin dose (i.e., 20 mU/min) that favored oxytocin.

#### Effect of Exogenous Oxytocin on Pain Relief

Two studies reported on pain relief.^73,74^ Odds ratios were computed at the median dose of oxytocin for each route of administration using full remission versus partial and full versus no remission to derive conservative estimates of effect. Patients with chronic low back pain who received the median dose (i.e., 100 μg/kg) of oxytocin intrathecally were more likely to report complete remission relative to placebo, with an odds ratio (OR) of 709.33 (95% CI 70.81, 7105.33).^74^ Similar effects were observed between oxytocin and placebo when administered intravenously (OR = 1.00; 95% CI 0.06, 16.52).^74^ Patients who experienced chronic migraines were also more likely to report remission following the intranasal administration of a 200 ng dose of oxytocin relative to placebo, with an OR of 27.44 (95% CI 5.31, 141.82).^73^

#### Endogenous Oxytocin Concentration Comparisons

Studies that compared plasma oxytocin levels in patients with chronic pain versus healthy controls were grouped as long as the association was reported independent of oxytocin administration.^61–63,66,73,74^

Table 4 presents a comparison of plasma oxytocin levels in healthy controls and patients with chronic pain. All studies reported significantly different (*p* < .01) basal plasma oxytocin levels between healthy controls and patients with chronic pain independent of pain condition. Wang et al.^73^ reported significantly higher mean plasma oxytocin levels in patients experiencing chronic migraine relative to healthy controls. Boström et al.^66^ observed the same trend in basal salivary oxytocin concentrations in patients with chronic migraine. It is noteworthy that only studies reporting on chronic migraine conditions observed this trend, while all other included studies observed significantly lower oxytocin concentrations among patients with chronic pain relative to healthy controls.^61,62,74^ Though not reported in the article, Anderberg and Uvnäs-Moberg asserted that the difference between plasma oxytocin levels in female patients with fibromyalgia and healthy controls were not significantly different (*P* = 0.55)^63^; however, the distribution of basal oxytocin levels in patients was larger than that in healthy controls.

**Table 4.** Mean endogenous oxytocin concentrations in chronic pain patients versus healthy controls.

	Plasma OT level (pmol/L)		
Study	Healthy controls (Mean ± SD)	Patients with chronic pain (mean ± SD)	Summary of results
Wang et al.^73^	9.43 ± 2.32	18.95 ± 4.83*	Higher plasma OT levels in patients
Boström et al.^66^	20.4 ± 1.7	44.2 ± 10.1*	Higher plasma OT levels in patients
Alfvén^61^	45.00 ± 15.42	30.50 ± 17.17*	Lower plasma OT levels in patients
Alfvén et al.^62^	63.00 ± 26.00	24.00 ± 15.00*	Lower plasma OT levels in patients
Yang^74^	28.10 ± 4.10	8.80 ± 3.40*	Lower plasma OT levels in patients
Anderberg and Uvnäs-Moberg^63^	NR	NR	The difference between plasma OT levels in HCs and PTs was not significantly different (*P* = 0.55). The distribution of plasma OT levels in PTs was larger than that in HCs

*Statistically significant difference between oxytocin levels in healthy controls and patients with chronic pain (*p* < .01).

HC = healthy control; NR = results were not reported, not able to be calculated or obtained; OT = oxytocin; PT = patient.

#### Effect of Exogenous Oxytocin on Depressed Mood

Three RCTs assessed symptoms of depressed mood through validated self-report measures and were considered similar; refer to Table 3. Mixed findings were observed with respect to the effect of oxytocin on depressed mood. The administration of oxytocin in Flynn et al.^68^ resulted in small improvements in depressed mood relative to placebo that were not statistically significant (*d* = 0.22; 95% CI −0.58, 1.02). Results from Mameli et al.^70^ favored the use of oxytocin for depressed mood, though this was not statistically significant (*d* = 0.05; 95% CI −0.71, 0.80), whereas Ohlsson et al.^71^ reported a small mean difference between oxytocin and placebo administration that favored placebo and was not statistically significant (*d* = −0.16; 95% CI −0.76, 0.44).

#### Effect of Exogenous Oxytocin on Anxious Mood

Three studies assessed the effect of exogenous oxytocin on self-reported anxious mood using validated scales. Flynn et al.^68^ observed that oxytocin administration resulted in a small reduction in anxious mood relative to placebo that was not statistically significant (*d* = 0.09; 95% CI −0.72, 0.88). Mameli et al.^70^ reported that the intranasal administration of oxytocin resulted in a small increase in anxious mood relative to placebo that was not statistically significant (*d* = −0.13; 95% CI −1.13, 0.39). Similarly, Ohlsson et al. reported that using oxytocin resulted in a small and nonsignificant increase in anxious mood relative to placebo (*d* = −0.15; 95% CI −0.45, 0.75).^71^

#### Association between Plasma Oxytocin and Anxious Mood

Two studies reported the correlation between plasma oxytocin concentrations and self-reported anxiety. Clark et al. reported no correlation between plasma oxytocin concentration taken during a resting state and anxiety ratings using Spearman’s correlation, *p* = 0.02.^67^ Anderberg and Uvnäs-Moberg^63^ measured levels of anxiety via daily symptom ratings on a 10-point numeric scale for 28 days. Mean scores for each participant were used to calculate the correlation against mean plasma concentrations from two blood tests. The authors reported a statistically significant moderate, negative correlation using Spearman’s correlation test, *p* = −0.46.

#### Effect of Exogenous Oxytocin on Adverse Effects

Five RCTs evaluated the effect of exogenous intranasal oxytocin administration on self-reported adverse effects relative to placebo.^68–72^ Reports of adverse effects were approximately equivalent between the oxytocin and placebo administration; refer to Supplemental Table 2.

## Discussion

The goal of this narrative review and meta-analysis was to triangulate evidence from diverse research to better understand the role of the oxytocinergic system on the experience of pain and emotional function among individuals who live with pain. Included studies provided evidence on (1) the effect of exogenous oxytocin on pain and emotional function, (2) the association between endogenous oxytocin and emotional functioning, and (3) basal oxytocin levels among patients with chronic pain relative to healthy controls.

### The Effect of Exogenous Oxytocin on Pain Intensity among People Who Live with Chronic Pain

Three studies reported on the effect of exogenous oxytocin administration on pain intensity that were sufficiently homogeneous for quantitative pooling. The pool effect was not statistically significant and favored oxytocin relative to control. This result is inconclusive and could reflect (1) lack of power due to a small number of trials; (2) the inclusion of patients with diverse chronic pain conditions. For example, relative to those who had no primary pain diagnosis, individuals with a diagnosis of irritable bowel syndrome who received oxytocin showed a reduction in abdominal pain^71^; and (3) methodological shortcomings among included trials. For example, Mameli et al.^70^ was limited by incomplete reporting of administration procedures, no formal evaluation of patient expectancy effects, and a small sample size (the interested reader should refer to the commentary by Rash and Campbell^78^).

Two RCTs reported on remission from pain and were subject to narrative review, indicating that the exogenous administration of oxytocin using a route that enters the central nervous system was significantly more likely to result in remission of pain than placebo among adults with low back pain and migraine.^73,74^ These results align with case studies that reported pain relief following intravenous oxytocin administration among two individuals with migraine^79^ and improvement in pain following the epidural administration of oxytocin among two older adults with pain secondary to cancer.^80^ Taken together, results are inconclusive, suggesting that additional rigorous trials are needed to determine the unbiased effect of exogenous oxytocin administration on pain among people who live with chronic pain.

### The Effect of Exogenous Oxytocin on Acute Pain Sensitivity

Three studies evaluated the effects of exogenous oxytocin administration on acute pain sensitivity among people who live with pain, with two trials reporting effects that favored oxytocin^65,69^ and one favoring placebo.^72^ Robust findings in Boll et al.^63^ may have been driven by their entirely male sample—this suggestion comes in light of secondary findings from Tracy et al.,^70^ who reported that oxytocin increased perceived intensity of noxious heat stimuli among female participants but not among males with chronic neck and shoulder pain (*d* = 0.71). These findings suggest that endogenous sex hormones may interact with oxytocin^81^ to influence pain perception among individuals with chronic pain conditions, with oxytocin being a more efficacious analgesic in males; refer to the Pertinent Debates subsection for further discussion.^72^

### Association between Endogenous Oxytocin and Pain Ratings

We observed a small, nonsignificant pooled correlation between endogenous oxytocin levels and pain ratings in our meta-analysis. It should be noted that two of the included studies assayed salivary oxytocin concentration in adults^66,67^ and one assayed plasma oxytocin concentration in children.^61^ Whereas Clark et al.^65^ reported a small negative association and included the largest sample size, Alfvén^59^ reported a moderate positive association and Boström et al.^64^ reported a large positive association. This heterogeneity raises the question of whether the chosen measures of oxytocin are adequately correlated with pain.

#### The Effect of Exogenous Oxytocin on Emotional Functioning

##### Depressed Mood

Narrative review of studies that evaluated depressed mood after oxytocin administration yielded inconsistent results. The ways in which oxytocin may act to alleviate depressed mood are difficult to disentangle given the cluster of depressive symptoms that often overlap with symptoms associated with chronic pain (e.g., hyperalgesias, fatigue and depressed mood involving dysregulation in the dopaminergic, serotonin, and oxytocin systems).^82,83^ Severity of depressed mood may also be a consideration. For example, Muin et al.^84^ reported that oxytocin improved self-reported depressed mood among mildly depressed women but not in more severe presentations.

##### Anxious Mood

Three RCTs evaluated the effect of oxytocin on self-reported anxiety, and narrative review produced inconclusive results. Mixed results are consistent with research in humans,^85^ with reports of both anxiogenic^86^ and anxiolytic properties.^87^ Moreover, it is possible that the dose of oxytocin administered was not strong enough to reach the necessary structures in the brain, the periphery, or the central nervous system^88^ to allow detectable improvement in mood.

### Association between Endogenous Oxytocin and Emotional Functioning

Results from meta-analysis indicated a small association between oxytocin levels and depressed mood that was not statistically significant.

### Basal Oxytocin in Chronic Pain Patients Relative to Healthy Controls

Oxytocin levels were observed to differ between individuals who experience chronic pain and healthy controls, and these differences appeared to differ based on chronic pain condition. It is important to note that differences have been observed in the bioanalytical method employed to quantify plasmatic oxytocin concentration, with values from radioimmunoassays differing widely from those of enzyme immunoassays.^89^ Different assay methods may capture molecules other than oxytocin and add to heterogeneity in assessment. Of interest, four studies included in this comparison used radioimmunoassay,^61,62,73,74^ with the remaining study using enzyme immunoassay.^66^ Taken together, results suggest that the oxytocinergic system may play a differential role in the development or experience of different pain diagnoses.

### Pertinent Debates

#### Sex Differences and the Effects of Oxytocin

Sex differences have been reported in previous research evaluating the association between oxytocin and pain and may explain heterogeneity in observed results. For example, research has reported consistent analgesia in healthy male volunteers after a single dose of oxytocin,^90,91^ including an RCT that reported results consistent with preclinical evidence for antinociceptive properties of oxytocin.^92^ Mixed results were reported in similar studies that included female participants,^90,93^ raising the question about whether women with primary pain diagnoses may be hypersensitive to painful stimuli. Related, estrogen has a priming effect on oxytocin synthesis, release, and receptor expression^94^ and has been observed to fluctuate during the menstrual cycle.^95^ More work needs to be done considering the suggestion that the association between oxytocin and pain may interact with endogenous sex hormones^72^ and that sex differences may be implicated in oxytocin mechanisms.^81,96^

#### Central versus Peripheral Effects of Oxytocin

Most biologically plausible mechanisms linking oxytocin and pain involve central availability. This is complicated in two ways: (1) two pathways of endogenous oxytocin release exist (one centrally and one peripherally) and (2) oxytocin is a peptide molecule that does not cross the blood–brain barrier.^11^ These factors have important implications for interpreting the results of this review. Specifically, research evaluating associations between oxytocin and pain relied on peripheral assays from saliva or plasma in all but one case. This is pertinent because the degree to which peripheral assays reflect central bioavailability is uncertain. The indices of endogenous oxytocin may also have influenced the reported levels, because several studies used measures of plasma oxytocin, whereas others used salivary oxytocin. Valstad et al.^97^ reported that blood plasma itself may not be an appropriate index of central oxytocin under resting conditions. Despite the appeal of using peripheral indices of oxytocin and the invasive nature of obtaining oxytocin concentrations from the cerebrospinal fluid, it is possible that levels of central oxytocin would vary more consistently with pain ratings. This is especially important given that Valstad et al. suggested that the presence of pain conditions may bias the coordination of central and peripheral oxytocin.^97^ Definitive conclusions regarding the assumption that single measures of endogenous oxytocin can index release of oxytocin in the brain have yet to be obtained.^98^

### Strengths

There are several strengths to this review. First, the present review represents the most comprehensive review to date on the influence of the oxytocinergic system on pain and function among those who live with pain. Second, we intentionally cast a broad net and reviewed diverse evidence through quantitative and narrative synthesis to triangulate the influence of the oxytocinergic system on pain and function among people who live with chronic pain. As such, we were able to gain insight into whether (1) the oxytocinergic system may be compromised among individuals who experience chronic pain relative to healthy controls and (2) exogenously administered oxytocin may have analgesic effects and improve pain and function. Third, this review has highlighted important gaps in our understanding and areas where future research is needed. Fourth, this review was preregistered, guided by clear clinical questions, included a robust systematic search that was developed in consultation with an independent information specialist and subject to peer review, and would be considered low risk of bias according to application of the AMSTAR 2 risk of bias assessment tool.^99^ Finally, authors of included studies were contacted to provide additional information about methodology and raw data in cases where reporting was not transparent.

### Limitations

There are several limitations inherent within the literature reviewed. First, heterogeneity in populations with chronic pain may have skewed the association between pain and endogenous oxytocin concentration. Most distinct, research on headache and migraine has been highlighted as an outlier in previous meta-analyses^97^ and has been flagged as containing unequivocal positive associations between oxytocin and pain^73,100^ when compared to other chronic pain studies. More specifically, oxytocin has been shown to be preferentially deposited in the trigeminal system,^101^ whose dysregulation has been associated with migraine.^102^ Second, the included studies often lacked adequate data to calculate the magnitude of the effects for the effect sizes of interest. This precluded us from investigating several outcomes of interest in meta-analysis. Third, two studies used peripheral exogenous administration as a proxy for central bioavailability, which may contribute to mixed findings regarding analgesic properties. Fourth, differences were observed in bioanalytic methods for quantifying plasmatic oxytocin concentration, which increases heterogeneity and complicates comparisons between studies. Fifth, Macdonald and Feifel^86^ asserted that there are several factors that might influence responses to oxytocin, such as gender, hormone status, genetic variations in the oxytocin system, and attachment history, that could confound consistent results. These pertinent confounds were often not considered in the analytic approach adopted within included studies. Finally, there was a scarcity of research reporting on pain-related interference or physical function as an outcome.

There are several limitations to the present review. First, diversity in populations with chronic pain sampled, methods for measuring pain and function, trial procedures, and methods of pain induction makes the included studies difficult to compare and limit interpretability. Second, heterogeneity impeded adequate or conclusive meta-analytic interpretation due to a lack of power. Third, the lack of a robust method to assess publication bias was a limitation when only three studies were meta-analyzed^103^; for this reason, additional studies would need to be included to ascertain the true impact of publication bias on the results. Fourth, we synthesized results across salivary and plasma (i.e., central and peripheral) indices of endogenous oxytocin among studies evaluating the association between pain and depressed mood. These routes follow different mechanistic pathways, and associations should be considered cautiously. Fifth, searches of the gray literature could have been beneficial in light of the scarcity of the current body of research.^99^ Finally, confidence in available evidence was not subject to formal GRADEing due to the limited and diverse literature included (i.e., all findings would be GRADEd very low confidence at this point).

### Future Directions

Evidence is needed from rigorous trials that examine the effect of exogenous administration of oxytocin among diverse populations with chronic pain to draw conclusions with greater precision and certainty. Our team is conducting an adequately powered, preregistered, double-blind, placebo-controlled multisite RCT investigating the effect of exogenous oxytocin administration on pain and pain-related interference among adults who experience chronic neuropathic, pelvic, and musculoskeletal pain (refer to Rash et al.^104^ for further details). The literature would also benefit from efforts to reduce risk of bias by reporting indices of precision of the estimate of the intervention or treatment effect to increase the interpretability of results. Finally, there is a need for future research to focus on two prominent areas of debate in the literature: (1) delineating sex differences that may confound results and (2) investigating complexities, mechanisms, and the intersection of central versus peripheral effects of oxytocin.

## Conclusion

A small number of heterogeneous studies were identified that evaluated the effect of oxytocin on pain and emotional function. Studies varied in design, populations with chronic pain evaluated, outcomes measured, and method of oxytocin administration and assessment. Results pertaining to the effect of exogenous oxytocin administration on pain were mixed but warrant further research in the form of rigorous clinical trials. Moreover, oxytocin levels differed between patients with chronic pain and controls, but directions differed by chronic pain condition. Current results point to potentially disparate associations between oxytocin and analgesia in patients with migraine relative to patients with other chronic pain conditions. Potential sex differences were also observed when investigating several outcomes. Taken as a whole, this is an area of investigation with true equipoise that is in need of additional rigorous trials that undertake more precise exploration of mechanisms of analgesic action.

## Supplementary Material

Supplemental Material
